# Low Hemoglobin-to-Red Cell Distribution Width Ratio Is Associated with Mortality in Patients with HBV-Related Decompensated Cirrhosis

**DOI:** 10.1155/2022/5754790

**Published:** 2022-02-14

**Authors:** Ze Yu, Tan Zhang, Jianjiang Shen

**Affiliations:** Department of Clinical Laboratory, Shengzhou People's Hospital, Shengzhou Branch of the First Affiliated Hospital of Zhejiang University, Shengzhou 312400, China

## Abstract

**Background:**

The prognostic role of hemoglobin-to-red blood cell distribution width ratio (HRR) in HBV-related decompensated cirrhosis (HBV-DeCi) has not been established. The present study is aimed at determining the potential of HRR as a predictive factor for the prognosis of HBV-DeCi patients.

**Methods:**

The study included 177 HBV-DeCi patients. The clinical outcome was death at 30 days. Multivariate regression analysis and receiver operating characteristic curve analysis were applied to assess the predictive value of HRR for poor outcomes.

**Results:**

A total of 26 patients (14.7%) had died by 30 days. Patients with unfavorable outcomes had lower HRR than patients with favorable outcomes. Multivariate analysis revealed that HRR and Model for End-Stage Liver Disease (MELD) score were independently associated with poor outcomes. Combination of HRR and MELD score may improve prognostic accuracy in HBV-DeCi.

**Conclusions:**

The present findings indicate that low HRR may be a promising predictor for mortality in HBV-DeCi patients.

## 1. Introduction

In China, hepatitis B virus (HBV) remains a leading cause of cirrhosis. The annual rate of progression from compensated cirrhosis to decompensated cirrhosis (DeCi) is approximately 3% [1, 2], with a 5-year mortality of 85% [3–5]. Liver transplantation is the only reliable life-prolonging intervention for DeCi. However, a shortage of donor livers and the substantial associated costs have limited its application. Therefore, accurate and early identification of high-risk patients has gained importance in clinical practice, because this may lead to adjustment of treatment strategies and help improve the clinical outcomes of HBV-DeCi patients.

In recent years, hematological parameters have been widely used for diagnosis and prognosis in liver diseases and have been paid increasing attention in clinical practice [6–9]. Red cell distribution width (RDW) and hemoglobin are indices derived from red blood cells that can reflect inflammation [10, 11] and malnutrition [12, 13]. Thus, it can be hypothesized that the combination of hemoglobin and RDW will provide an objective and readily available prognostic indicator for certain clinical problems. Recently, hemoglobin-to-RDW ratio (HRR) has been identified as an accurate and novel prognostic indicator in several malignant diseases, with low HRR linked to poor outcomes in affected patients [14–19]. However, the role of HRR for prognosis of HBV-DeCi patients remains to be established. Therefore, the present study is aimed at exploring the clinical utility of HRR as a prognostic predictor of short-term mortality in patients with HBV-DeCi.

## 2. Materials and Methods

### 2.1. Patients

**Figure 1:**
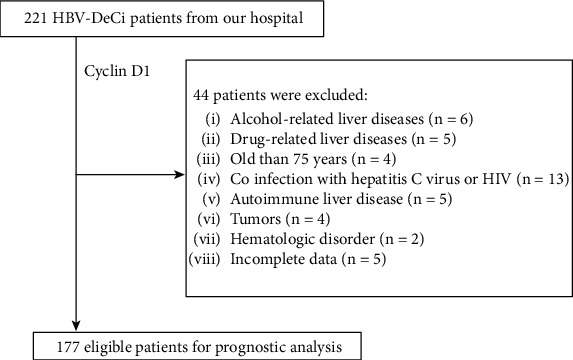
Flow chart of the enrolled participants.

We retrospectively reviewed data for HBV-DeCi patients aged ≥18 years who were admitted to our hospital from May 2019 to September 2021. All patients presented with clinical manifestations of decompensated liver disease for the first time. The inclusion criteria were as follows: age < 75 years and HBV surface antigen positivity > 6 months. The exclusion criteria were as follows: (1) history of other liver diseases, such as autoimmune, alcohol-related, or drug-related liver diseases; (2) coinfection with hepatitis C virus or HIV; (3) malignant tumor; (4) hematologic disorder; (5) blood product transfusion or immunomodulatory therapy in past 3 months; and (6) incomplete medical data. DeCi was defined by the development of at least one among variceal bleeding, ascites, hepatorenal syndrome, jaundice, or encephalopathy [20]. Finally, 177 patients were enrolled (Figure 1). Patients received antiviral therapy and supportive treatment after hospitalization and were followed up for at least 30 days to identify short-term mortality. This study was approved by the Institutional Ethics Committee.

### 2.2. Data Collection

The following data were collected at the time of admission: age, sex, and laboratory variables (total bilirubin, total protein, albumin, creatinine, blood urea nitrogen, aspartate aminotransferase [AST], alanine aminotransferase [ALT], international normalized ratio [INR], hemoglobin, RDW, platelet count). INR was measured using a CA-1500 autoanalyzer (Sysmex, Kobe, Japan). Hemoglobin, RDW, and other blood cells were measured using an XE-2100 autoanalyzer (Sysmex). ALT, AST, and other serum biochemical parameters were measured using a 7600 clinical analyzer (Hitachi, Tokyo, Japan). Anemia was defined as hemoglobin < 130 g/L (men) or 120 g/L (women) in accordance with the recommendations from the WHO [21]. HRR was defined as hemoglobin (g/L) divided by RDW (%). Liver disease severity was evaluated by the Model for End-Stage Liver Disease (MELD) score as previously described [22].

### 2.3. Statistical Analysis

Baseline data were expressed as median (interquartile range) for continuous variables and number for categorical variables. Differences in variables were compared using the Mann–Whitney *U* test or chi-square test, as appropriate. The correlation between HRR and MELD score was determined by Spearman rank correlation analysis. Univariate and multivariate analyses were performed to determine risk factors for 30-day poor outcomes. The entry and removal probabilities for the step-wise method were set to 0.05 and 0.10, respectively. Area under the receiver operating characteristic curve (AUC) was calculated to estimate and compare the predictive values of HRR and MELD score. The statistical analyses were carried out using SPSS19.0 or MedCalc 12.7 software. For all statistical analyses, *P* < 0.05 was considered significant.

## 3. Results

### 3.1. Study Population

We identified 177 HBV-DeCi patients who met the inclusion criteria. The most common type of decompensation or complication in our cohort was ascites (*n* = 138; 78.0%), followed by variceal bleeding (*n* = 40; 22.6%), hepatorenal syndrome (*n* = 18; 10.2%), and hepatic encephalopathy (*n* = 8; 4.5%). The median HRR was 6.32 (interquartile range, 4.87 to 7.70) at admission. The correlation analysis revealed that HRR was negatively correlated with MELD score (*r* = −0.179; *P* = 0.017).

A total of 26 participants (14.7%) died within 30 days. The causes of death were hepatic failure (*n* = 7), gastrointestinal bleeding (*n* = 5), encephalopathy (*n* = 6), and hepatorenal syndrome (*n* = 8). As shown in Table 1, significant differences were observed for total protein, creatinine, INR, MELD score, total bilirubin, hemoglobin, RDW, HRR, and blood urea nitrogen between the survivors and nonsurvivors.

### 3.2. Risk Factors for Adverse Outcomes

**Figure 2:**
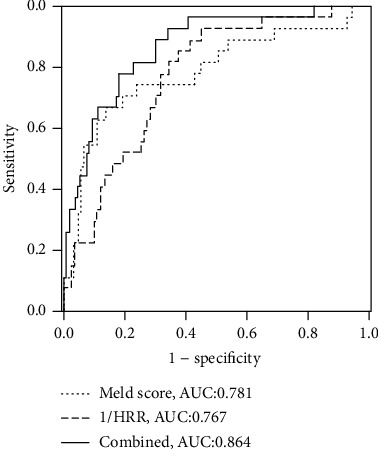
ROC curves for MELD score and 1/HRR used alone or in combination to predict mortality in HBV-DeCi patients.

The potential risk factors for adverse outcomes identified in the univariate and multivariate analyses are shown in Table 2. The univariate analyses showed that hemoglobin, total protein, RDW, HRR, and MELD score were correlated with prognosis for 30-day mortality. In multivariate regression analysis adjusted for other indicators, HRR and MELD score remained associated with adverse outcomes. Next, ROC curve analyses were conducted to assess the relative efficiencies of HRR and MELD score to predict poor outcomes. HRR was changed to 1/HRR by an inverse transformation. The cutoff values were 17.6 for MELD score (sensitivity, 66.7%; specificity, 86.0%) and 6.01 for HRR (sensitivity, 85.2%; specificity, 62.7%). For prediction of mortality, the AUC of HRR was 0.767 and slightly lower than the AUC of MELD score (0.781; *Z* = 0.184; *P* = 0.854). Furthermore, when 1/HRR and MELD score were combined, the AUC increased to 0.864 (Figure 2).

### 3.3. Clinicopathological Characteristics in High and Low HRR Groups

The participants were stratified into two groups according to the cutoff value for HRR: low group (HRR ≤ 6.01) and high group (HRR > 6.01). As shown in Table 3, low HRR was significantly associated with increased mortality, RDW, MELD score, and INR and decreased total protein, albumin, and hemoglobin.

## 4. Discussion

Early identification of HBV-DeCi with the risk of adverse outcomes is an important task in clinical practice. We investigated the value of HRR as a prognostic indicator for mortality in these patients. Our results showed that reduced HRR was correlated with poor survival, and that HRR can serve as a simple prognostic indicator for unfavorable outcomes. Currently, the MELD score is the most commonly used scoring system to assess illness severity and predict mortality in end-stage liver disease. The MELD score is determined by three conventional parameters (INR, total bilirubin, and creatinine) and requires complex calculations that are inconvenient for routine practice [22]. Our results demonstrated a negative correlation between HRR and MELD score and indicated that the predictive power of HRR was comparable to that of MELD score. However, HRR is based on only two routine blood tests, making it more convenient and simpler to calculate than the MELD. In addition, combining HRR with MELD improved the prognostic accuracy to 0.864.

There are two reasons that may partially explain the mechanism for how HRR influences the prognosis of HBV-DeCi patients. First, the results revealed that hemoglobin was decreased in nonsurvivors compared with survivors. Anemia is known to be extremely common in liver disease patients, and its presence is linked with poor outcomes including liver decompensation, liver failure, and increased risk of mortality [23]. Some complicated mechanisms for the involvement of anemia in liver diseases have been described, including folate and vitamin B12 deficiencies, bone marrow suppression, renal insufficiency, and variceal bleeding [24, 25]. Among the 177 patients in the present cohort, 150 (84.7%) suffered from anemia. These results are consistent with the reported prevalences of 50%–87% in liver disease patients in previous studies [26–29]. Recently, Cai and colleagues reported that hemoglobin level may help to improve risk stratification and can be considered an independent risk factor for prognosis in HBV-DeCi patients [30]. However, our multivariate analysis indicated that hemoglobin failed to predict mortality in the present cohort. Second, our results showed that RDW was higher in nonsurvivors than in survivors. RDW is an index for the heterogeneity of erythrocytes and can help to determine possible causes of anemia in clinical practice [31]. Furthermore, it has been reported to show diagnostic and prognostic potential in a variety of disorders, including liver diseases [32]. For example, Lou and colleagues demonstrated a relationship between RDW and HBV in patients at various clinical stages and observed that high RDW was linked to mortality in these patients [33]. In ensuing studies, Gianni and colleagues found that RDW can help to predict the risk of 1-month unfavorable outcomes in patients with acute DeCi [34]. Although the exact mechanism for how high RDW is linked to worse survival in liver disease patients remains unclear, it is generally considered that the increase in RDW is partly due to changes in erythrocyte maturation caused by inflammation [35]. Prior studies indicated that inflammation may influence bone marrow function. Under inflammatory conditions, red blood cell maturation may be suppressed, and thus newer and larger reticulocytes may enter the peripheral blood, resulting in an increased RDW [10, 36]. Other studies identified RDW as an inflammation-based marker, and inflammation is considered to play a critical role in the development and progression of liver diseases [37, 38]. Similar to hemoglobin, RDW was not found to be an independent predictor of mortality in our multivariate analysis. It is possible that hemoglobin and RDW are both influenced by several factors, including inflammation [11], aging [39, 40], and malnutrition [41]. Because HRR is a ratio, it may represent a more effective and stable indicator than hemoglobin or RDW alone. We found that low HRR was correlated with parameters that reflected liver disease severity and also correlated with high mortality. We further found that low HRR was mainly caused by increased RDW and decreased hemoglobin. Therefore, we propose that HRR reflects the nutritional status and inflammatory condition and may be useful to predict the prognosis of HBV-DeCi patients. Of course, further studies are required to determine the underlying mechanism.

In summary, HRR is a simple and readily available biomarker in clinical practice that can provide auxiliary prognostic information for poor outcomes in HBV-DeCi patients. Two limitations of the study are its small sample size and the lack of an external validation cohort. Thus, the present findings should be further verified by prospective studies.

## Figures and Tables

**Table 1:** Patient characteristics at baseline.

	All patients (*n* = 177)	Surviving patients (*n* = 150)	Nonsurviving patients (*n* = 27)	*P*
Gender (female/male)	34/143	26/124	8/19	0.182
Age (years)	53.0 (46.0-62.3)	52.5 (46.0-62.0)	55.0 (49.0-64.5)	0.252
Total protein (g/L)	61.5 (56.1-66.9)	61.6 (57.0-67.3)	56.4 (50.9-65.3)	0.014
Albumin (g/L)	31.1 (27.1-34.7)	31.2 (27.3-34.8)	30.1 (26.5-32.9)	0.309
ALT (U/L)	31.0 (17.0-55.8)	30.0 (17.0-53.0)	41.0 (16.3-63.8)	0.423
AST (U/L)	46.0 (28.0-74.0)	46.0 (28.0-72.8)	56.0 (29.0-135.8)	0.310
Serum creatinine (*μ*mol/L)	72.0 (59.0-86.3)	71.0 (59.0-83.0)	92.0 (67.8-124.0)	0.002
Total bilirubin (*μ*mol/L)	42.0 (19.0-117.0)	36.5 (18.0-99.0)	78.0 (51.0-239.0)	0.009
Blood urea nitrogen (mmol/L)	5.60 (4.20-7.50)	5.20 (4.10-6.78)	8.20 (6.50-14.00)	<0.001
INR	1.37 (1.18-1.64)	1.33 (1.18-1.58)	1.64 (1.27-1.93)	0.003
Hemoglobin (g/L)	104.0 (86.0-121.0)	108.0 (90.0-121.0)	95.0 (70.5-110.3)	0.011
RDW (%)	16.3 (15.0-18.5)	15.9 (14.8-17.8)	20.1 (17.2-21.6)	<0.001
HRR	6.32 (4.87-7.70)	6.57 (5.24-7.82)	4.86 (3.94-5.76)	<0.001
Platelet (×10^9^/L)	69.0 (43.8-112.5)	70.0 (43.0-112.0)	71.0 (54.3-122.5)	0.744
MELD score	11.7 (6.9-17.4)	11.4 (6.5-15.8)	20.3 (13.1-22.6)	<0.001

Data are expressed as number or median (interquartile range). Abbreviations: ALT: alanine aminotransferase; AST: aspartate aminotransferase; INR: international normalized ratio; RDW: red blood cell distribution width; HRR: hemoglobin-to-red cell distribution width ratio; MELD: Model for End-Stage Liver Disease.

**Table 2:** Logistic regression analyses to identify risk factors associated with mortality in patients with HBV-DeCi.

	Univariate			Multivariate		
	Odds ratio	95% CI	*P*	Odds ratio	95% CI	*P*
Total protein (g/L)	0.926	0.877-0.977	0.005			
MELD score	1.978	1.106-1.296	<0.001	1.206	1.104-1.317	<0.001
HRR	0.574	0.438-0.752	<0.001	0.552	0.401-0.759	0.001
Hemoglobin (g/L)	0.974	0.956-0.993	0.006			
RDW (%)	1.362	1.186-1.565	<0.001			
Blood urea nitrogen (mmol/L)	1.032	0.992-1.074	0.123			
Age (years)	1.023	0.985-1.062	0.235			

Abbreviations: CI: confidence interval; MELD: Model for End-Stage Liver Disease; HRR: hemoglobin-to-red cell distribution width ratio; RDW: red blood cell distribution width.

**Table 3:** Clinical data according to HRR values.

	Low group (HRR ≤ 6.01, *n* = 78)	High group (HRR > 6.01, *n* = 99)	*P*
Gender (female/male)	15/63	19/80	0.853
Age (years)	53.0 (46.0-63.0)	54.0 (46.0-62.0)	0.699
Total protein (g/L)	59.9 (53.7-63.7)	62.0 (57.8-67.9)	0.008
Albumin (g/L)	30.1 (26.4-32.6)	31.6 (28.2-35.5)	0.037
Total bilirubin (*μ*mol/L)	58.0 (17.0-117.0)	38.0 (19.5-116.8)	0.587
Blood urea nitrogen (mmol/L)	6.20 (4.18-8.22)	5.10 (4.15-6.90)	0.056
INR	1.45 (1.25-1.73)	1.30 (1.16-1.54)	0.008
Serum creatinine (*μ*mol/L)	73.0 (59.0-92.0)	72.0 (59.3-85.0)	0.349
Platelet (×10^9^/L)	66.0 (39.0-123.0)	74.0 (49.0-111.8)	0.637
MELD score	14.3 (9.1-18.6)	11.3 (6.5-15.0)	0.020
Hemoglobin (g/L)	85.0 (72.0-96.0)	111.0 (108.0-130.0)	<0.001
RDW (%)	18.5 (17.0-20.8)	15.2 (14.2-16.1)	<0.001
30-day mortality (yes/no)	22/56	5/94	<0.001

Data are expressed as number or median (interquartile range). Abbreviations: HRR, hemoglobin-to-red cell distribution width ratio; INR, international normalized ratio; MELD, Model for End-Stage Liver Disease; RDW, red blood cell distribution width.

## Data Availability

The data are available upon reasonable request.

## Abbreviations

ALT: Alanine aminotransferase
AST: Aspartate aminotransferase
AUCs: Areas under the curve
CI: Confidence interval
DeCi: Decompensated cirrhosis
HBV: Hepatitis B virus
HRR: Hemoglobin-to-red cell distribution width ratio
INR: International normalized ratio
MELD score: Model for End-Stage Liver Disease score
RDW: Red blood cell distribution width
ROC: Receiver operating characteristic.
